# Novel Concept of Micro Patterned Micro Titer Plates Fabricated via UV-NIL for Automated Neuronal Cell Assay Read-Out

**DOI:** 10.3390/nano11040902

**Published:** 2021-04-01

**Authors:** Mirko Lohse, Manuel W. Thesen, Anja Haase, Martin Smolka, Nerea Briz Iceta, Ana Ayerdi Izquierdo, Isbaal Ramos, Clarisa Salado, Arne Schleunitz

**Affiliations:** 1 Micro Resist Technology GmbH, Köpenicker Str. 325, 12555 Berlin, Germany; m.thesen@microresist.de (M.W.T.); a.schleunitz@microresist.de (A.S.); 2 Joanneum Research Materials, Institute for Surface Technologies and Photonics, 8160 Weiz, Austria; anja.haase@joanneum.at (A.H.); Martin.Smolka@joanneum.at (M.S.); 3 TECNALIA, Basque Research and Technology Alliance (BRTA), Mikeletegi Pasealekua 2, 20009 Donostia-San Sebastián, Spain; Nerea.briz@tecnalia.com (N.B.I.); ana.ayerdi@tecnalia.com (A.A.I.); 4 Innoprot, Parque Tecnológico de Bizkaia, Edificio 502, Primera Planta, 48160 Derio-Bizkaia, Spain; iramos@innoprot.com (I.R.); csalado@innoprot.com (C.S.)

**Keywords:** nanoimprint lithography, R2R UV-NIL, neuronal cell assay

## Abstract

The UV-nanoimprint lithography(UV-NIL) fabrication of a novel network of micron-sized channels, forming an open channel microfluidic system is described. Details about the complete manufacturing process, from mastering to fabrication in small batches and in high throughput with up to 1200 micro titer plates per hour is presented. Deep insight into the evaluation of a suitable UV-curable material, mr-UVCur26SF is given, presenting cytotoxic evaluation, cell compatibility tests and finally a neuronal assay. The results indicate how the given pattern, in combination with the resist, paves the way to faster, cheaper, and more reliable drug screening.

## 1. Introduction

Cell assays are an essential part of biochemical research, pharmaceutics and diagnostics. The interaction between cells and towards external stimulus, such as a potential drug, can give insight into the potential toxicity or pharmaceutic effect of such chemicals [1,2,3,4,5]. Some of the cell-based assays to determine the toxic potential of drugs are reactive oxygen species measurements (ROS) [6], Caspase activation [7], life cell imaging, [8] DNA damage [9], and neurite outgrowth [10]. Traditionally, such cell assays have been performed on flat surfaces using commercially available microtiter plates (MTP) fabricated with thermoplastic materials like polystyrene (PS), polymethyl methacrylate (PMMA) or cyclo-olefin copolymers (COC). However, it has been shown that the surface topography can play a crucial role in the tendency of cells to adhere to the surface [11], grow in defined directions or shapes [12], and even, in terms of stem cells, in which way cells differentiate [13,14,15]. While most of these patterns range from a few microns to several tens of microns [16,17,18,19,20], several examples show that even nanosized features [21,22,23] can influence the cell behavior. One particular class of cells of great interest is neurons. Their use in drug screening has been reported extensively and different commercial devices are available [24]. The toxic effects of the drugs on neurons could be measured by analyzing the neuron projection characteristics (neurites), such as neurite average length, neurite root number and neurite branching. On flat state-of-the-art microtiter plates the entanglement and overlapping of the different neurites make automated read out difficult and unreliable. However, there is a multitude of different ways for improving here. While some use micro or nano patterned surfaces to help cells to orientate them along the pattern [25,26], one common way is to block neuron cell cores with a physical barrier, which only the neurites can pass [27,28,29,30]. Thereby, the reaction of single neurites to external stimulus can be observed. We believe that with a well-chosen patterned surface, the deviations appearing during the analyses of flat surface neuron clusters can be reduced and automated read-out can be improved. Looking at the literature, most examples of patterned surfaces rely on a thermal process, either by injection molding or thermal nanoimprint lithography (NIL) [31], limiting the choice of material and the functionality beyond the one given by the pattern. UV-curable materials and the use of UV-NIL offer the chance to broaden the range of materials that can be used and incorporate intrinsic functionalities such as hydrophilicity of the material or functional groups on the cured surface. Further, UV-NIL is a highly scalable process. It was shown that by UV-NIL both nano- and micro-featured patterns can be replicated and even whole lab-on-a-chip devices [32].

**Scheme 1:**
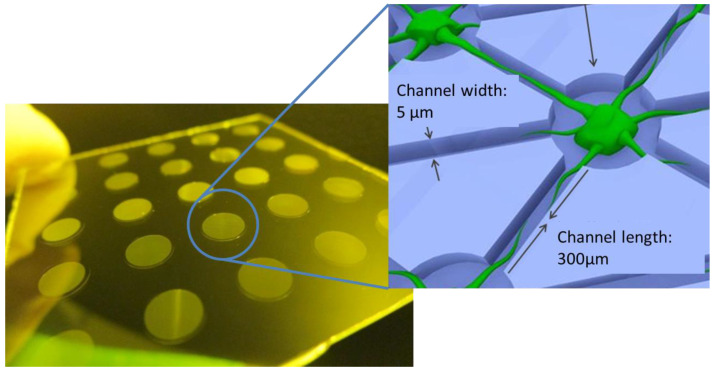
Micro patterned bottom foil of a micro titer plate and schematic design of the underlying concept of cell separation.

In our approach, we want to overcome the limitations of standard cell assays on flat surfaces, by proposing to employ micropatterning processes for an innovative micro titer plate design (a) driving the growth of neurites to targeted 2-dimensional channels and thereby avoid crowding and entanglement of neurons and protecting them from removal during washing; and (b) limiting the number of neurons in a given spot, making image analysis more feasible and reliable (see Scheme 1). Further, the connection of each well by the 2-dimensional channels creates an open channel microfluidic system that enables selective functionalization of the channel walls at the base area of each well and channel. Our aim for this work is to show that UV-NIL is a highly feasible method to fabricate such patterns in high volume by roll-to-roll (R2R) fabrication. Therefore, we have chosen a UV-curable resist that is known for its compatibility with continuous R2R fabrication and show its compatibility with the demands of cell testing [33].

## 2. Materials and Methods

### 2.1. Mastering

Silicon wafers from Siegert Wafer GmbH (Aachen, Germany) were used and oxygen plasma treated (900 W, 1 min) before coating. Negative type photoresist SU-8 3000 was received by Kayaku Advanced Materials Inc. (Westborough, MA, USA) and soft mold UV-PDMS KER-4690 was bought from Shin-Etsu Chemical Co., Ltd. (Tokio, Japan). The mr-Dev 600 and OrmoStamp was offered by micro resist technology GmbH (Berlin, Germany). The 1H,1H,2H,2H-Perfluorooctyl-trichlorosilane (CAS number [78560-45-9], F13-OTCS) was received from ABCR GmbH (Karlsruhe, Germany) and used without further purification.

Plasma activation of the silicon wafer was done on a PS300 Semi Auto oxygen plasma oven from PVA Tepla AG (Wettenberg, Germany). Polymer masters were fabricated using a MA6 mask aligner from Süss MicroTec SE (Garching, Germany). Optical micrographs of imprinted structures were recorded using a BX51M microscope from Olympus K.K. (Tokio, Japan) equipped with a Color View Soft Imaging System. SEM images were performed on an Inspect F50 from FEI Company (Hillsboro, OR, USA).

**Figure 1:**
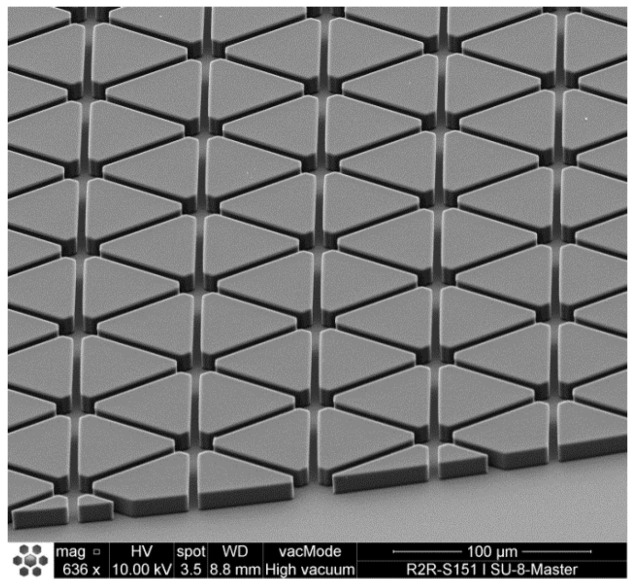
SEM image of the SU-8 polymer master showing the microfluidic network. At the bottom, parts of the feeding channel are visible.

**Scheme 2:**
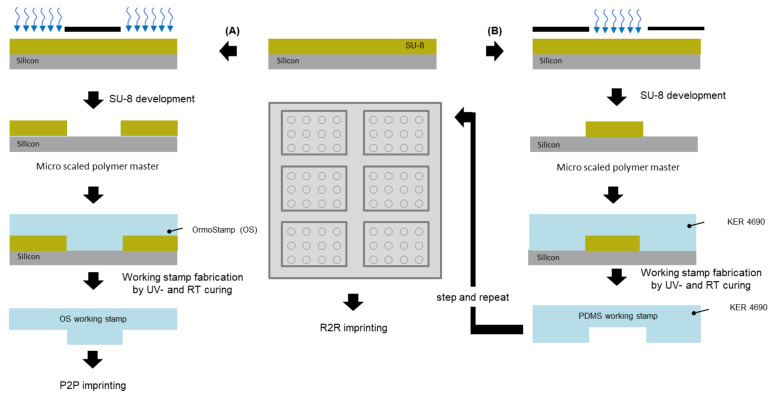
Schematic representation of the fabrication process to obtain the micro patterned bottom foil either by plate-to-plate (P2P) (**A**) or roll-to-roll (R2R) (**B**).

The fabrication of thick SU-8 based negative type photoresist polymer masters (see Scheme 2) has been published in detail before [34]. Briefly, a 17 µm thick SU-8 3000 layer was patterned with the corresponding mask on a MA6 mask aligner and developed using mr-Dev 600. Afterwards, an antisticking layer (ASL) was applied via chemical vapour deposition (CVD) of F13-OTCS following a standard procedure derived from literature [35]. Two different orientations (A and B in Scheme 2) of lithography masks were used depending on the final use, resulting in either recessed or free-standing microfluidic channels. Exact horizontal side wall accuracy could be achieved with the established photolithography process (see Figure 1). It is noteworthy that a full 96 well plate could not be mastered as this exceeded the dimension of a 6-inch wafer, which was the limit of the applied mask aligner. Nevertheless, a master with approximately 60 micro titer wells was fabricated.

Orientation A with free trenches was directly transferred into a hard-working stamp typically applied in plate-to-plate imprint processes with extremely high requirements on pattern fidelity. Therefore, in a reverse-NIL process a defined amount of OrmoStamp [36] was poured on the ASL equipped polymer master and covered with a glass backplate. After the liquid resin had spread and filled the cavities of the master, the photo-polymerization process was initialized by UV-exposure in the MA6 mask aligner (D = 2000 mJ/cm^2^). Afterwards, a post exposure bake (150 °C, 30 min) and coverage with a CVD F13-OTCS ASL (similar to above) gave the final OrmoStamp working stamp used as such for the plate-to-plate (P2P) imprinting. Working stamps with sizes up to 7 × 7 cm^2^ (25 micro titer wells) have been fabricated and used for batch imprinting. With larger working stamp sizes the forces needed to separate the rigid polymer master from the rigid glass like OrmoStamp were too high and resulted in destruction of the polymer master.

Orientation B, with free standing trenches, was used for the fabrication of a soft stamp intermediate for the production of large-scale shims for the R2R manufacturing. Therefore, a defined amount of silicone prepolymer, enough to cover the full wafer, was poured onto the polymer master with orientation B. Once the cavities of the SU-8 were completely filled with the KER-4690, which was monitored via microscope imaging, the UV-PDMS was exposed in the MA6 mask aligner (D = 2000 mJ/cm^2^) to activate the crosslinking. The detachment of the UV-PDMS stamp copy from the SU-8 master was conducted 12 h after exposure and storage at room temperature. The process of replicating SU-8 polymer masters with PDMS is well known and has already been applied for the manufacturing of other microwell applications using generic PDMS [37,38,39]. With this approach a full 6″ wafer was replicated and used for the step and repeat fabrication for the R2R shims.

### 2.2. P2P UV-NIL Imprints

Foil substrates PET (Melinex, 125 µm) used for P2P and R2R test imprints were purchased from Pütz Folien. Foils were precleaned with isopropanol and, if needed, activated by a short oxygen plasma (200W, 45 s). UV-NIL resist mr-UVCur26SF was offered by micro resist technology GmbH (Berlin, Germany). mr-UVCur26SF is a solvent-free and fast curing acrylate formulation designed for R2R and Inkjet applications [33]. Its high pattern stability, high double bond conversion (>95%), low shrinkage (about 8%) and low auto fluorescence made it an ideal candidate for the given application.

Two different ways for processing the P2P imprint have been performed. The precleaned and preactivated foil was either spin coated in a manual tabletop spin coater at 3000 rpm for 60 s to achieve a thin layer of resist or a film was prepared using a 10 µm thick doctor blade. For imprinting, the film was brought into contact with a working stamp bearing the negative of the desired pattern. By manually laying the stamp from one side of the imprint area to the other, a slow filling of the cavities was achieved. In terms of the microscopic pattern aimed at in the publication, the filling of the pattern could be observed through an optical microscope. When complete filling was achieved, curing of the UV-curable resist was either conducted using a tabletop device (CNI v.2.0 supplied by NIL Technology ApS, Lyngby, Denmark) or using an MA6 from Süss MicroTec (Garching, Germany). Optical micrographs of the imprinted structures were recorded using a BX51M microscope from Olympus K.K. (Tokio, Japan) equipped with a Color View Soft Imaging System.

### 2.3. Converting to R2R UV-NIL

Continuous roll-to-roll (R2R) UV-NIL has been proven to be an efficient way to fabricate microfluidic devices [32]. The shim fabrication for large area imprinting derived from the previously described PDMS replica of the SU-8 polymer master (orientation B, Scheme 2). Those 6″ working stamps were used in a proprietary step and repeat process by Inmold ApS (Horsholm, Denmark) [40], followed by the sputtering of a layer of chromium and aluminum, 3–4 nm and 50 nm, respectively, at the premises of Joanneum Research. Finally, the shim was coated with an ASL to minimize the releasing energies during high throughput fabrication [41]. The obtained finished shim was mounted on a magnetic roller inside the R2R machine with a minimal seam between the two ends. The R2R imprint experiments were realized on a customized Basecoater R2R-UV-NIL imprinting machine from Coatema Coating Machinery GmbH (Dormhagen, Germany). The resist was applied via a double column gravure coating resulting in a comparable thick film of resist (approximately 20 µm, not optimized) on a PET-foil and the pattern was imprinted using the metalized roller shim. Web speeds between 0.1 m/min and 2 m/min were tested and curing was conducted using an LED-source (395 nm).

Final imprints were investigated for air bubble defects by standard optical microscope. 

### 2.4. Cytotoxicity Validation

In this paper, we have used several cell lines provided by INNOPROT (Derio, Spain); kidney tubular epithelial cells (KTEC, Cat N: P10647), conjunctive epithelial cells (Epit. conj., Cat N: P10870), Dermal endothelial cells (Skin. Endo., Cat N: P10861), hepatic endothelial cells (Hepatic. Endo., Cat N: P10652), and two cell lines obtained from ATCC (Virginia, USA); bone osteosarcoma epithelial cells (U2OS, Cat N: ATCC HTB-96) and embryonic kidney 293 cells (HEK293, Cat N: ATCC CRL-1573).

Biocoating and cell seeding was performed manually. Visualization of cells and neurons was done using Zeiss Axio Observer Fluorescence microscope equipped with Apotome 2 system (structured illumination system by Zeiss, Germany).

To investigate the compatibility of the used UV-NIL, resist cytotoxicity tests following UNE-EN-ISO 10993-5: 2009 have been conducted. Briefly, following the processing guidelines of the resist, a layer of the material was applied on PET foil (precleaned with isopropanol) via spin coating (3000 rpm, 2 acc, 30 sec) resulting in a 700 nm thick film, followed by curing under an inert (CO_2_) atmosphere in a MA6 mask aligner (D = 2000 mJ/cm^2^). A defined surface area of the cured resist was then placed in contact with an incubation medium (complete Eagle’s minimal essential medium (EMEM)) for 24 h at 37 °C (surface area to sample volume was defined as 6 mL/cm^2^). 

The American Type Culture Collection CCL 171 cell line was cultured in EMEM medium supplemented with 10% fetal bovine serum (FBS) and 1% antibiotic penicillin/streptomycin (under standard cell-culture conditions (37 ± 2 °C and of 5% CO_2_) until the cells reached the confluent state. For the assay 1 × 10^4^ CCL-171 cells were seeded in complete EMEM in 96-well plates and incubated at 37 °C and 5% CO_2_. The following day, cells were incubated with the sample extracts (100, 75, 50 and 25%) and control extracts for 24 h. Cell viability was quantified by means of the WST-1 test. WST-1 reagent is added to each well and 4 h later the optical density of this solution in each well is measured at a wavelength of 450 nm using a microplate reader (BioTek Instruments, Inc. (Winooski, VT, USA), Powerwave XS). Each measurement was conducted five times in parallel. Additionally, a negative control, a positive control and a blank were included in the assay. As for the ISO guidelines, high density polyethylene was used as the negative control and polyurethane film containing 0.1% zinc diethyldithiocarbamate (ZDEC) as the positive control.

According to ISO 10993-5: 2009, a sample is considered toxic if cell viability at the highest concentration analyzed (namely, 100%) is below 70% of viability. In this test the overall performance of different materials can be tested in terms of impurities that can migrate from the bulk into the incubation medium. It is noteworthy, that no material properties, such as its cell adhesion properties were investigated at this stage. Further, no material related toxicity (e.g., certain functional groups on the surface) was evaluated at this stage.

To investigate the biocompatibility of the used UV-curable material, cell assays were conducted on a cured plain film, which was treated with plasma, or used pristine, as well as coated with different types of biopolymers (collagen and poly-D-lysine, PDL). Tested cell types were kidney tubular epithelial cells (Monkey KTEC, P10647 Innoprot), conjunctive epithelial cells (human Epit conj, P10870 Innoprot), hepatic endothelial cells (human Hepatic endo, P10652 Innoprot), skin endothelial cells (human skin endo, P10861), bone osteosarcoma epithelial cells (human U2OS, HTB-96 ATCC) and human embryonic kidney 293 cells (human HEK, CRL-1573 ATCC). Cell lines were tested on their cell adhesion as well as proliferation on the given surface at 24 h and after 72 h being in contact to the material surface (incubation temperature 37 °C). For such assay, 20,000 cells/well were cultivated on the given substrate and after a specific time (24 h and 72 h), the cell viability is determined by WST-8 colorimetric method. At the end of the desired incubation period WST-8 reagent (Cell Counting Kit-8, Dojindo) was added. Cells were then incubated at 37 °C for 60 to 90 min at 37 °C in the dark. The absorbance of the formazan product at the wavelength of 450 nm was quantified using a microplate reader. The data was expressed as a percentage of viable cells compared to the survival of a control group (untreated cells). If the given material surface treatment resulted in a cell viability of 80% or higher compared to a control (pristine PS), the cured material passed the test and was considered non-toxic for this cell line.

### 2.5. Neuronal Growth

For the neuronal cell assays PMMA foil (100 µm) was used as a substrate. Bottomless micro titer plates (material polystyerene) were bought from Greiner and double sided tape were purchased from BiFlow Systems GmbH.

Double sided duct tape was applied by lamination to the bottomless MTP and circular cut-outs were obtained via laser cutting using a picosecond laser (MicroStruct Vario, 3D Micromac AG, Chemnitz, Germany) with a wavelength of 355 nm. Biocoating and cell seeding was performed manually. Visualization of cells and neurons was done using Zeiss Axio Observer Fluorescence microscope equipped with Apotome 2 system (structured illumination system by Zeiss, Oberkochen, Germany).

Neuronal cell assays were performed on the patterned surface to evaluate the improvement of neurite outgrowth in the produced devices. They were fabricated in a reverse NIL process on PMMA as substrate. A defined amount of liquid resin was applied on the stamp and brought into contact with the substrate. After a complete filling was observed under the microscope, the resist was cured on a CNI 2.0 tool (365 nm, I = 33 mW/cm^2^, D = 2000 mJ/cm^2^). The PDL coated patterned foil was glued to the bottom of a 96 well micro titer plate by commercial double sided tape, and used for neuronal cell growth tests. 

Rat cortical neurons, (Innoprot Cat N: P10102) at 50 k cells/well cell density were placed on the surfaces and incubated up to 72 h with neurobasal culture medium (05790 Stemcell Technologies, Vancouver, Canada) supplemented with NeuroCult SM1 (05711, Stemcell Technologies). Then cell bodies were immunostained with anti-beta III Tubulin-Alexa 488 antibody (AB15708A4, Sigma-Aldrich, St. Louis, MO, USA) and nuclei with DAPI (D9542, Sigma-Aldrich, St. Louis, MO, USA) following the manufacturer’s indications. Images were analyzed by confocal microscopy and processed using Wimasis-Wimneuron software (Onimagin Technologies, Cordoba, Spain).

## 3. Results and Discussion

### 3.1. Mastering

As described above. the micro patterned bottom foil of a micro titer plate was designed in a way that a 1000 µm thick feeding channel is placed around a network (see Figure 1) of singular micro cavities (diameter 40 µm) connected to each other by microchannels (length 140 µm, 5 µm wide). Microfluidic simulation showed that the rounded edges on the interconnection between channel and micro cavity enabled a faster and more homogenous filling of the resulting microfluidic network. For the micro patterned bottom in the microcavities, a pattern height of 17 µm was found to be optimal between fast production cycles of the pattern and spatial separation of the bottom of the micro titer plate from the triangular plateaus in order to maximize the optical output and minimize internal interfering signals.

### 3.2. P2P UV-NIL Imprints

A hard working stamp derived from the orientation A (see Scheme 2) was used to replicate the pattern into the low viscose UV-NIL resist. Details on cytotoxicity and cell compatibility were tested and are discussed in detail later. 

**Figure 2:**
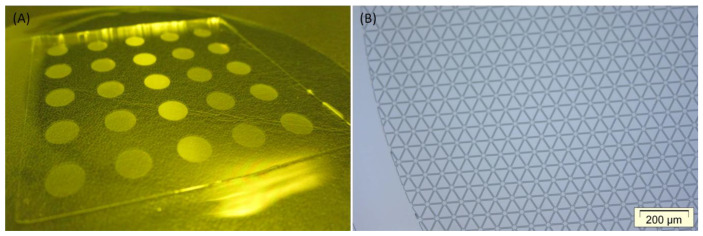
Imprint on PMMA via P2P-imprinting with a hard working stamp. (**A**) Photograph of the imprint and (**B**) microscope image showing the microfluidic network.

The replication of the desired pattern worked well and defect-free imprints were obtained using PMMA as the substrate (see Figure 2). Processing the resist via spin coating resulted in a too thin film (approximately 700 nm at 3000 rpm) for the required pattern height. Hence, other deposition methods had to be tested. As most promising and most comparable to the high-throughput R2R process, a 10 µm thin film was applied via doctor blading. The so obtained film of uncured resist was manually brought into contact with the working stamp. Under a microscope the filling of the microscopic cavities could be observed. It is noteworthy that filling time s comparatively high due to large aspect ratio of the desired structure and the limited occurrence of capillary forces. With such large patterns, the process step of filling becomes increasingly demanding in time as the capillary forces that help filling small features sizes only play a minor role. Further, the volume of air that needs to be removed and replaced by resist in such large micron sized features is significantly larger compared to nano patterns resulting in an even slower process. However, the low viscosity of the material enables a faster absorption of the entrapped air, in comparison to other high viscose resists. Nevertheless, in the case of the tested pattern, filling times of roughly 10 min were observed. One advantage of the R2R based approach, besides the potential high volume production, is the continuous filling and curing during imprinting. With a sufficiently well-designed shim, trapped air can be avoided and “pushed out” by the liquid resist front on the roller [42].

**Figure 3:**
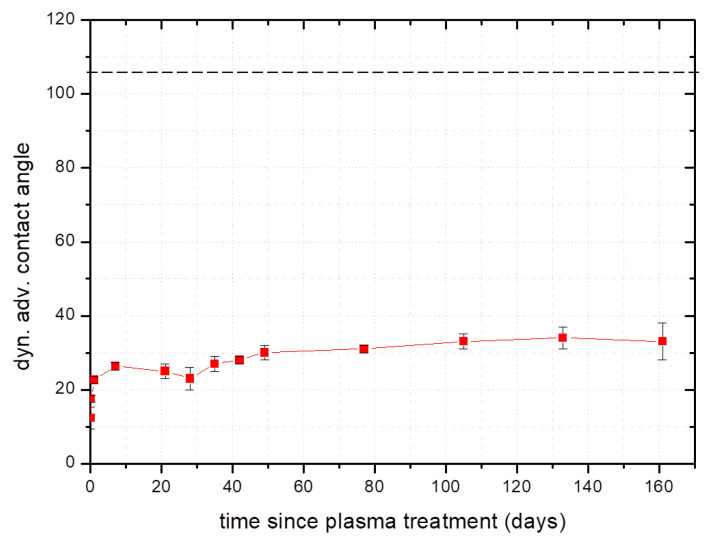
Advancing dynamic water contact angle (WCA) of a cured film after a short oxygen plasma treatment and its development over time. The dashed line indicates the initial adv. dyn. WCA of 107° before plasma treatment.

The obtained imprints were used to conduct the initial filling tests of the open microfluidic channels via capillary forces. This was a necessary prerequisite for functionalization of the cavities and channels with a neuron growth-enabling substance such as fibronectin. It was observed that with a structure height of 17 µm, spontaneous filling of the channels and cavities with aqueous solution was possible for sufficiently hydrophilic surfaces. In the case of the used UV-NIL material, the low surface tension of the resist prevented spontaneous channel filling. However, it was found that a short oxygen plasma treatment (100 W, 10 s) was sufficient to increase the hydrophilicity of the cured resist and spontaneous channel filling was obtained. Nonetheless, it could be shown that such short plasma was sufficient to keep the selectivity of filling only the channels with aqueous solutions and thus paving the way for subsequent biofunctionalization. This surface hydrophilization was shown to be stable for several weeks after an initial regain in hydrophobicity (measured by advancing water contact angle, see Figure 3). After this increase, the contact angle of around 30° was stable for 3 months (details on the measurement of the dynamic water contact angle can be found in the supporting information). The slow increase can be explained by the high network density preventing a rearrangement of hydrophobic groups to the surface. Even though this trend was only investigated for a few months, it is highly likely that the cured film will never reach its initial hydrophobicity and will stay hydrophilic.

### 3.3. Converting to R2R UV-NIL

**Scheme 3:**
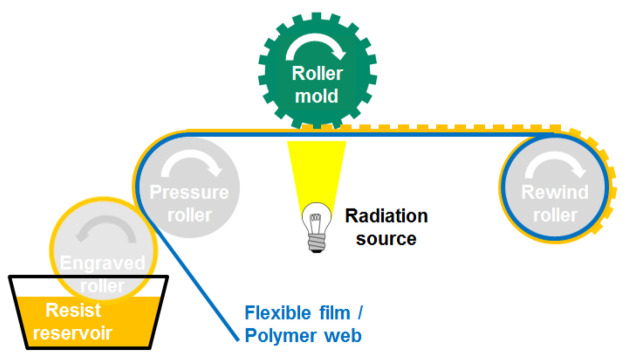
Schematic representation of the R2R fabrication process.

Roll-to-roll (R2R) imprinting is a continuous fabrication method in which a film of UV-curable material is applied on a flexible substrate and patterned via a roller based shim (see Scheme 3).

**Figure 4:**
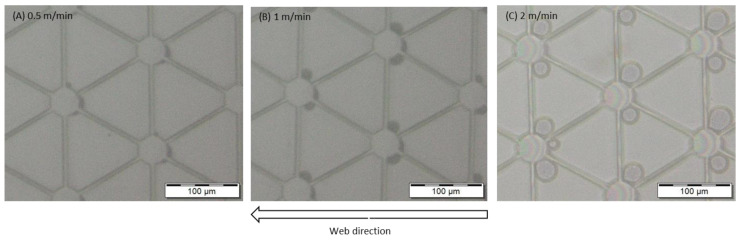
Microscope images of the patterned surface after R2R UV-NIL imprinting showing the defect rate depending on the imprint speed ((**A**) 0.5 m/min; (**B**) 1 m/min; (**C**) 2 m/min).

Similar to the P2P UV-NIL imprints, avoiding the entrapment of air and giving enough time to fill the pattern were crucial to obtain a defect free imprint. Hence, the comparatively slow roller speeds of only up to 2 m/min were applied and visual inspection under a light microscope of the imprinted foils suggested a defect free imprint at speeds below 1 m/min. As seen in Figure 4 and Figure S1, with an increasing web speed the amount of air defects increased. At 0.5 m/min almost no entrapped air was visible, while at 2 m/min a high defect rate could be observed. The entrapped air bubbles were placed on the last corner of the triangles, where they were pushed together. With increasing web speed, the amount of entrapped air increased, as shown in the microscope images. In comparison to other less demanding patterns, the chosen design did not allow a complete pushing out of the air and only at speeds up to 1 m/min was the absorption of the entrapped air fast enough for a defect free imprint. It is noteworthy that other resists with higher viscosity were tested as well, but no defect free imprints were obtained. With the optimized shim design, patterned foils for around 1200 full micro titer plates can be imprinted in one hour showing the potential for mass fabrication.

### 3.4. Cytotoxicity Validation

For a device to be used in contact with cells or neurons, it needs to be proven to be non-cytotoxic. Following ISO10993-2005 mr-UVCur26SF is to be considered non-cytotoxic under the mentioned curing conditions. However, since the resist is a photocurable material and the used photoinitiator shows a dependence on wavelength and intensity, the curing properties might alter depending on the given fabrication method. Further, impurities resulting from a rub off of the stamp used (in the case of the R2R fabrication, an aluminum coating was used), might interact with the cell lines and create a cytotoxic behavior of the cured surface. We tested an assay of different batches of the resist, as well as different curing conditions (see Table 1) to demonstrate the broad manufacturing window of our material.

It can be seen that the resist can be cured either by broad band mercury irradiation as well as specific LED wavelength. Further, the intensity can be varied in a range between 10–50 mW/cm^2^ and the dose can be optimized between 500 and 2000 mJ/cm^2^. Within this broad range of curing conditions none of the samples showed cytotoxic behavior leaving a large process window in which the pattern can be fabricated.

**Figure 5:**
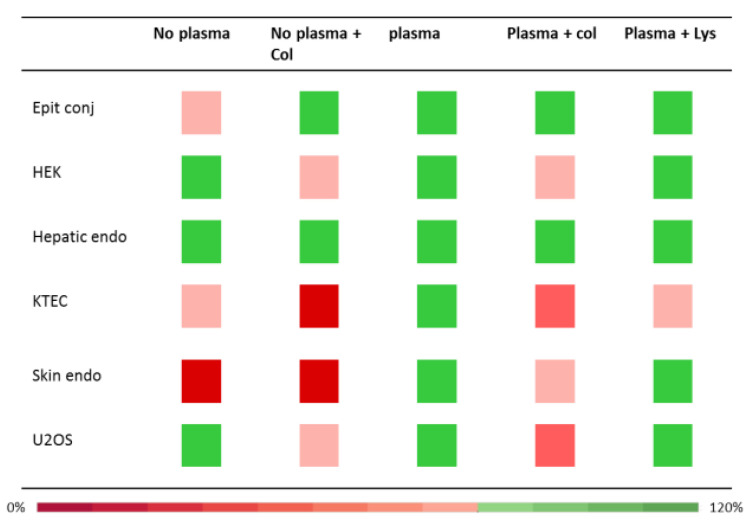
Summary of the cell viability tests of different cell lines after 72 h on a flat surface of mr-UVCur26SF under different coating conditions. The color shift from red to green indicate the increasing viability, with 80% (light green) being sufficient for further testing.

Further, the compatibility of the tested material with different cell lines: kidney tubular epithelial cells (KTEC), conjunctive epithelial cells (Epit. Conj.), hepatic endothelial cells (Hepatic. Endo.), dermal endothelial cells (Skin. Endo.), bone osteosarcoma epithelial cells (U2OS) and embryonic kidney 293 cells (HEK293) has been tested, both on the pristine cured resist and in combination with different biocoatings (lysine and collagen). The cell lines were chosen to show the broad applicability of the mr-UVCur26SF. The cell lines in the investigation contained both tumoral cells (U2OS, HEK) and primary cells. Within the primary cells, they included epithelial and endothelial cells. In addition, there were cells from different sources, monkey, mouse and human. The results were compared to pristine PS, a standard material used for micro titer plates in state of the art assays. Any sample with a viability of 80% or higher was considered as compatible with the cell line. The results are summarized in a qualitative fashion in Figure 5 and Figure S2. A more detailed quantitative analysis is given in the supporting information. Most cell lines showed an overall good compliance with the material. After 72 h, all showed a cell viability that was comparable to state-of-the-art cell assay materials when the surface was treated with short oxygen plasma. Poly-D-lysin showed the best performance of the two tested biocoatings and it could be seen that besides KTEC, all cell lines were compatible with a lysine coating. Collagen on the other hand showed some significant differences in cell viability when used directly or in combination with initial plasma. A clear image for these findings is still under investigation. However, it appears that one major reason might be the polarity of the resist after plasma treatment and resulting rearrangements of the biocoating. Further, it is known that the hardness of the substrate can have an influence on the adhesion strengths of different cells towards the substrate. Both material’s properties can influence the compatibility of the resist towards the given cell lines.

The results shown should function as an indicator to which cell types the mr-UVCur26SF is compatible. It is noteworthy that an incompatibility towards a specific cell lines does not automatically indicate a toxicity. The given test assay rather indicates the tendency of the cells to adhere on the resist surface. For further tests, a surface functionalization with a short plasma treatment, followed by poly-D-lysine (PDL) treatment was chosen.

### 3.5. Neuronal Growth

**Figure 6:**
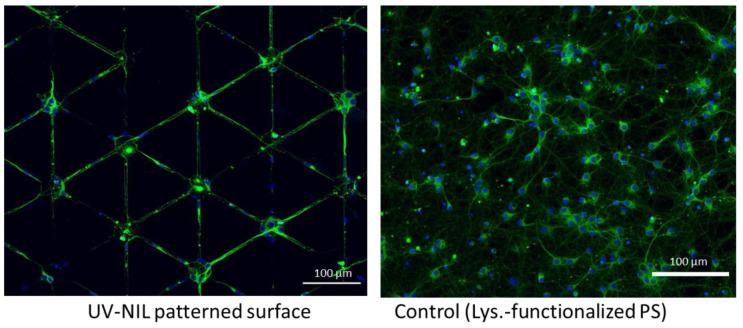
Confocal microscope image of seeded neurons on a patterned surface of the used UV-NIL material (**left**) and an unstructured PS micro titer plate (**right**) for reference. Neurites (cell bodies are stained with Anti Tubulin III-488 antibody (green) and nuclei with DAPI (blue)) aligned along the microfluidic channel.

The behavior of neurons in contact to the patterned surfaces was the main objective of these investigations and the compatibility was tested accordingly. The patterned samples were compared to standard unpatterned polystyrene 96 MTP covered with PDL. As can be seen in Figure 6, the neuronal cells aligned well along the channels of the microfluidic system (left panel) and were much better separated in comparison to the flat surface of the standard MTP (right panel). The majority of the neurons were placed within the microfluidic network as the plateaus were not covered with PDL and hence were less adhering for the cells (data not shown). Moreover, using confocal microscopy only, the fluorescent signal from the cells within the patterned structures could be monitored. The selectivity of the cells to attach in the coated areas gave an increased control over the separation, allowing a more organized culture. The overall number of neuronal cells decreased in comparison with the flat PS surface. This simplified the further imaging analysis. Via confocal fluorescence microscopy, single cells could be observed and their neurite outgrowth studied. With these promising results neuroprotection assays have been conducted to test the improvement of our approach over current protocols.

**Figure 7:**
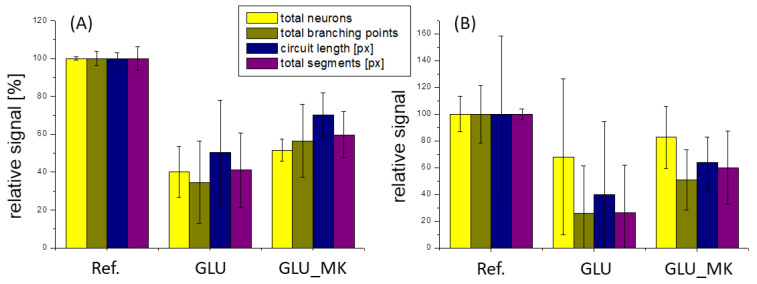
Influence of a neurotoxin (glutamate, GLU) on the total neuron count, total branching points, circuit length and total segments for a patterned surface (**A**) and a flat PS surface (**B**). Additionally, the results with a neuron protective agent (MK-801, GLU_MK) are shown.

For such assay, neuronal cells were cultured in neurobasal growth medium in the absence and presence of a neuroprotective compound, MK-801 (Catalog number M-107, Sigma-Aldrich, St. Louis, MO, USA) at 10 μM concentration. Then, 100 µM glutamate solution was added to provoke injury to cells. After 15 min the glutamate was replaced by fresh medium and thoroughly washed. 48 h later, cells were immunostained with Anti-Tubulin III-488 antibody and imaged on a confocal microscope. Control wells were also prepared with neither glutamate nor MK-801 incubation. Different parameters of neuronal growth, such as the total number of vital neurons, length of neurites, total branching points or others were studied. The data shown in Figure 7 and Table S1 are the average of three independent studies, in which individual wells were seated with the same number of cells, treated in the same way and quantified individually.

The results indicate that neurons react to the external stimulus of glutamate and MK-801 similarly, both on the flat PS surface (Figure 7B) and on the patterned surface (Figure 7A). In contact with the neuronal cell toxin (glutamate), the number of viable neurons, their branching points, circuit length and total segments decreased, as expected. This loss in viability can be partially overcome using a neuron protective (MK-801). While it is difficult to compare the results from the flat PS surface with the one patterned with a UV-NIL material, as essential parameters such as total cell count and cell density differ dramatically, a few crucial findings can be made. Firstly, the overall trend in both cases in terms of damage and recovery of the neurons was similar when using patterned surfaces in comparison with standard plate protocol. A decrease in viability was observable when the neurons came in contact with the toxin and a certain protection from this toxin could be achieved using an appropriate reagent. This indicates that the neurons within the trenches stay in contact with the medium above the micro grooves and reagents can migrate towards the cells. This can be considered as an improvement because it allows the use of lower cell numbers for the same result. Secondly, the conformal stress that was put on the cells, needing to adjust themselves towards the pattern, did not result in a higher sensitivity for neuronal toxins. Thirdly, and most importantly, the reduced number of cells, and thereby easier automated “assignment” to specific cells led to a reduced deviation in all investigated parameters. The total number of cells on the patterned surface was only approximately a third in comparison to the PS reference. However, the deviation in cell numbers was reduced from 13% to 0.8%. The branching points decreased by a factor of approximately 6.5 between the patterned and unpatterned surfaces, while the deviation was 3.5% in comparison to 21.6%. The total circuitry length was about 2.2 times shorter than on the flat surface, but the deviation was 2.7% in comparison to 61% of the unpatterned surface. For the total segment length decreased by a factor of roughly 5.6, the deviation stayed almost the same for both cases. Similar results could be observed for the samples treated with the neurotoxin and the neuron protective agent. In the supporting information the absolute values are given from which the relative numbers were derived. While we are aware that this assay can only function as a proof-of-principle and more data need to be created to reduce the deviation shown in the results, we believe that the pattern we suggest can help to create more accurate automated read-out systems by reducing the total neuron count in a defined area and thereby also reducing the entanglement. Given this, better reliability is expected between experiments and therefore the analysis would be more robust. Further, it can be seen that a standard protocol for analyzing the behavior of neurons towards specific drugs can be easily adopted towards the patterned surface without significant changes or needs for optimization. In addition, this organized arrangement will allow other types of applications and experiments apart from neurite outgrowth or even with other types of cells.

## 4. Conclusions

In this paper, we have shown a straight forward approach to decrease the standard deviation of a neuronal cell assay by separating cell clusters with a micro patterned surface of a standard micro titer plate for easy read-out. Using industrial compatible processes, we have fabricated polymer shims and working stamps to manufacture such patterned surfaces both in a small scale P2P fashion as well as continuous high throughput R2R fabrication. Furthermore, we have evaluated the cytotoxicity and cell compatibility of our chosen material, the fast curing mr-UVCur26SF, for a multitude of different cell lines and curing conditions, showing the high compatibility of the material, even beyond the scope of the publication. Finally, initial neuronal cell assay tests were conducted, comparing the chosen patterned surface with a flat polystyrene reference, showing that our initial idea of spatial separation of neurons prevents clustering to some extent and reduces the overall standard deviation of the preformed assay. We believe that with our approach a new class of neuron based drug screening assays can be developed, leading to faster and more reliable high-throughput drug screening.

## Figures and Tables

**Table 1:** Summary of different tested curing conditions and the resulting nontoxicity (following UNE-EN-ISO 10993-5: 2009) of mr-UVCur26SF. Standard deviation for all samples was below 5%.

Light Source	λ (nm)	Intensity I (mW/cm^2^)	Dose D (mJ/cm^2^)	Cell Viability (%)
Hg	UV	10	2000	97 ± 4
Hg	UV	20	500	101 ± 5
Hg	UV	20	1000	99 ± 5
Hg	UV	20	2000	95 ± 3
LED	365	10	2000	93 ± 4
LED	365	20	500	96 ± 4
LED	365	20	1000	91 ± 6
LED	365	20	2000	96 ± 4
LED	365	30	2000	91 ± 4
LED	365	50	2000	91 ± 4
LED	390	20	2000	97 ± 4
LED (R2R) ^1^	395	-	-	93 ± 5

^1^ Applied roller speed 1 m min^−1^.

## Supplementary Materials

The following are available online at https://www.mdpi.com/article/10.3390/nano11040902/s1, Figure S1: comparison of a low viscous (15 mPas) UV-NIL resist with a higher viscous, Figure S2: unpatterned surface of mr-UVCur26SF: (a) Pristine; (b) collagene coated on pristine surface; (c) O_2_ plasma treated surface (d) collagene coated on O_2_ plasma treated surface (e) poly-D-lysine coated on O_2_ plasma treated surface, Table S1: Average values of two separate sets of experiments with the given deviation in their results.
