# Supplementary material for: Novel Concept of Micro Patterned Micro Titer Plates Fabricated via UV-NIL for Automated Neuronal Cell Assay Read-Out
Source: Nanomaterials (Basel). 2021 Apr 1;11(4):902. doi: 10.3390/nano11040902 (PMC8065385; doi:10.3390/nano11040902)
Supplement: Supplementary file 1 [file nanomaterials-11-00902-s001.pdf]

nanomaterials

Supplementary Materials

Dynamic water contact angle

Evaluating the advancing dynamic water contact angle was achieved using an instrumental setup where a water droplet can be placed on the surface by a µL-syringe,, which stays in contact with it. As the volume is slowly increased, a video of the process is made. After the measurement, the recorded video is analysed frame by frame and in a semi-automated fashion, the water contact angle is calculated from an idealized circle. For each surface, at least two droplets were measured and at least 5 points were taken per droplet. From those an average contact angle was calculated.

Converting to R2R UV-NIL

As described in the main text an increased defect rate during R2R imprinting was observed when the web speed reached a certain threshold. This limit is defined by the given pattern and strongly influenced by the viscosity of the imprint material. Supporting this finding in figure S1 a comparison of a low viscous (15 mPas) UV-NIL resist with a higher viscous (approx. 650 mPas) is shown. The tendency of bubble defect formation is quite clear.

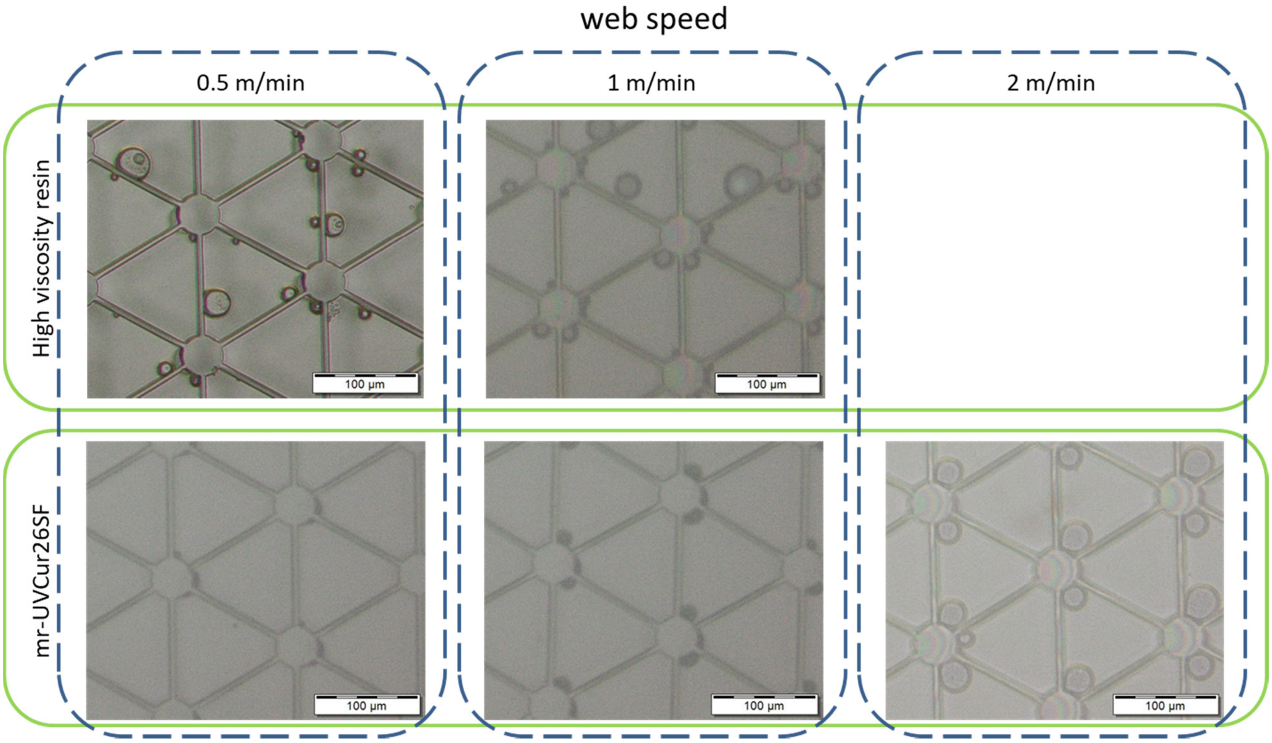

Figure S1. Comparison of a low viscous (15 mPas) UV-NIL resist with a higher viscous.

Cell compatibility

As described in the main text different cell lines were tested for their compatibility towards mr-UVCur26SF. Therefore, cells (20000cells/well) were seeded on a flat surface of the material and their viability was tested after 24 and 72h. Each measurement was repeated twice and for each measurement two samples were taken. The results are qualitatively shown in the main text of the manuscript. Full data with standard deviation are shown in figure S1. The cell lines have been chosen to investigate a broad range of cells. These cells contained both tumoral cells (U2OS, HEK) and primary cells. Within, the

primary cells, they include epithelial and endothelial cells. In addition, there are cells from different sources, monkey, mouse and human.

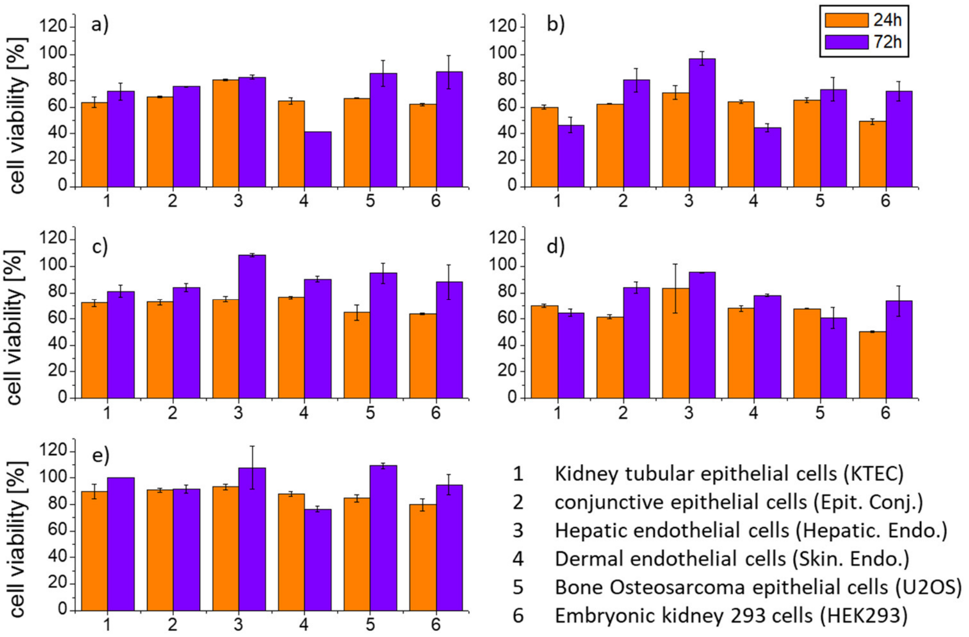

Figure S2. unpatterned surface of mr-UVCur26SF: (**a**) Pristine; (**b**) collagene coated on pristine surface; (**c**) O_2 plasma treated surface (**d**) collagene coated on O_2 plasma treated surface (**e**) poly-D-lysine coated on O_2 plasma treated surface.

Neuron cell assay

In the main text the relative results of the performed neuronal cell assay using glutamate (GLU) as a neuron toxin and MK-801 as a neuron protective agent are shown. In table S1 the absolute values as the average of three separate testings are shown from which the figure 7 derived. Those results are the average of three independent wells seeded with the same number of cells, treated in the same way, and quantified independently.

Table S1. Average values of two separate sets of experiments with the given deviation in their results.

	Total Neurons	Total Branching Points	Circuitry Length [px]	Total Segments [px]
**Cur26_LYS**	84,5 ± 0,7	114 ± 4	29200 ± 800	290 ± 18
**Cur26_LYS_GLU**	34 ± 11	40 ± 25	15000 ± 8000	120 ± 57
**Cur26_LYS_GLU_MK**	44 ± 5	65 ± 20	21000 ± 3000	180 ± 35
**Control**	250 ± 33	740 ± 160	65000 ± 40000	1620 ± 65
**GLU**	170 ± 144	200 ± 260	26000 ± 40000	430 ± 600
**GLU_MK**	204 ± 57	375 ± 170	42000 ± 10000	970 ± 400
